# Draft Genome Sequences of *Chromobacterium* Strains Isolated from Water Systems in Central Western Brazil

**DOI:** 10.1128/mra.00417-22

**Published:** 2022-09-26

**Authors:** Raylane Pereira Gomes, Renata Kikuda, José Daniel Gonçalves Vieira, Debora de Jesus Pires, Christopher Dunlap, Lilian Carla Carneiro

**Affiliations:** a Institute of Tropical Pathology and Public Health, Federal University of Goiás, Goiânia, Goiás, Brazil; b State University of Goias, Campus Morrinhos, Goiás, Brazil; c United States Department of Agriculture, National Center for Agricultural Utilization Research, Crop Bioprotection Research Unit, Peoria, Illinois, USA; University of Delaware

## Abstract

We report the draft genome sequences of four *Chromobacterium* strains. This report includes the draft genome sequences of four environmental strains, isolated from surface waters in Brazil.

## ANNOUNCEMENT

The genus *Chromobacterium* is Gram-negative, heterotrophic, saprophytic bacteria, causing occasional infections, being opportunistic pathogens, often fatal, with high metabolic diversity. These microorganisms are useful for industry and various applications, the main one being the ability to produce a purple pigment, called violacein, that has many biotechnological applications (1 to 3). Recently, *Chromobacterium* strains have been reported with insecticidal activity, useful against many important insect pests (4, 5). According to the List of Prokaryotic names with Standing in Nomenclature (https://lpsn.dsmz.de/), the genus *Chromobacterium* has 14 taxa with valid nomenclature.

The four strains were isolated for this study. Samples (250 μL) of raw surface water from aquatic environments (Table 1) were spread on two media, MacConkey and R2A agar, in triplicate. The plates with the culture medium and the samples were incubated for up to 48 h. After the incubation period, it was observed that only four colonies, one from each aquatic environment, had purple/purple characteristics of colony coloring, were circular, and were Gram-negative, bacilli, or coccobacilli. These were isolated for this study, as they are suggestive of the genus *Chromobacterium*.

**TABLE 1:** Isolation and genome characteristics of four C*hromobacterium* strains isolated from samples from water systems in Central Western Brazil

Identification	Chromobacterium vaccinii CR1	Chromobacterium violaceum CR2	Chromobacterium piscinae CR3	Chromobacterium vaccinii CR5
Isolation culture medium	MacConkey agar	R2A agar	R2A agar	R2A agar
Origin	Raw surface water	Raw surface water	Raw surface water	Raw surface water
Date isolation	27 November 2017	27 November 2017	20 July 2017	13 December 2018
Geographic location	16°34'30.54″S; 49°13'55.02″O	16°28'25.05″S; 49°6'43.87″O	18°01'39.3″S; 49°22'13.0″W	16°54'16.3''S; 49°07'37.8''W
Water environment of isolation	João Leite stream, Goiânia, Goiás, Brazil	João Leite stream, Goiânia, Goiás, Brazil	Buriti lake, Goiatuba, Goiás, Brazil	Meia Ponte river, Goiânia, Goiás, Brazil
Size (bp)	4,850,677	4,836,175	4,959,806	4,861,385
GC content (%)	64.5	65	63.3	64.41
*N*_50_ (bp)	265,153	262,734	205,164	205,164
L50	8	7	7	35
No. of contigs	75	49	51	228
No. of subsystems	319	334	328	324
No. of coding sequences	4,679	4,761	4,850	4,995
No. of RNAs	85	94	87	78
GenBank accession no.	JAJNRT01	JAJMMA01	JAJNRU01	JAJNRV01
Hyperlink to the publicly available data record	https://www.ncbi.nlm.nih.gov/assembly/GCF_021083355.1/	https://www.ncbi.nlm.nih.gov/assembly/GCF_020991315.1/	https://www.ncbi.nlm.nih.gov/assembly/GCF_021083375.1/	https://www.ncbi.nlm.nih.gov/assembly/GCF_021083405.1/

The four bacterial strains were cultivated in LB broth and agar, and they were stored in 20% glycerol in a freezer at −20°C. Genomic DNA was extracted (6) with modifications (7). The genomic DNA was prepared for sequencing using a Nextera XT library preparation kit, following the manufacturer’s suggested protocols. The prepared libraries were sequenced using a MiSeq DNA sequencer using the MiSeq V3 2 × 300 sequencing kit.

All processing and assembly of the genomes was performed in CLCbio Genomics Workbench 21.0.4 and under default parameters, unless stated. The sequencing reads were quality trimmed using the 0.05 quality limit, and reads below 50 bp were removed, using CLCBio Genomics Workbench. The genome draft was assembled based on 30% overlap between sequencing reads/contigs that shared at least 95% nucleotide identity. Annotation was performed using NCBI Prokaryotic Genome Annotation Pipeline using the best-placed reference protein set (GeneMarkS-2+; software version 5.3) (8, 9).

Genome comparisons and phylogenetic trees were made using BIGSdb software (8). The phylogenetic tree was constructed using MEGA X software (10). Neighbor-joining trees were reconstructed using the Tamura-Nei model (11) with a gamma correction (alpha value = 0.5); this model was chosen on the basis of the likelihood test implemented in MEGA X. Measures of bootstrap support for internal branches were obtained from 1,500 pseudoreplicates. A data set of genes identified as the core genome of the selected strains was found using BIGSdb software (12) and used as the basis of the phylogenetic analysis.

**FIG 1:**
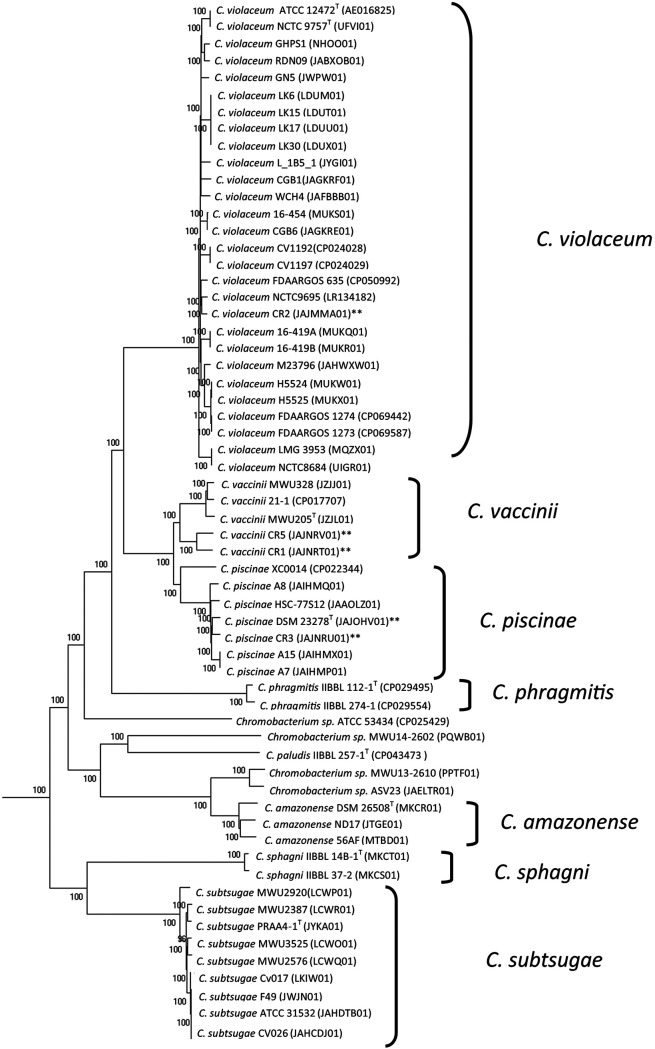
Core genome phylogenetic analysis of the genomic sequencing of four *Chromobacterium* strains isolated from samples from water systems in central western Brazil. Strains marked with ** were sequenced in this study. Details of the isolation characteristics and genomic 72 sequencing of the strains are described in Table 1.

The four strains were identified as demonstrated in the genomic tree (Fig. 1). The four strains isolated, CR1 was identified as Chromobacterium vaccinii (4,850,677 bp, 64.4% GC content and 75 contigs), CR2 was identified as Chromobacterium violaceum (4,836,175 bp, 65% GC content and 65 contigs), CR3 was identified *as* Chromobacterium piscinae (4,959,806 bp, 63.3% GC content and 51 contigs), and CR5 was identified as Chromobacterium vaccinii (4,861,385 bp, 64.41% GC content and 228 contigs).

Details of the isolation characteristics and genomic sequencing of the strains are described in Table 1.

### Data availability.

The genome sequences described in these studies were deposited in GenBank with the accession number JAJNRT01 to *C. vaccinii* CR1, JAJMMA01 to *C. vaccinii* CR5, JAJNRV01 to *C. piscinae* CR3, and JAJNRU01 to C. violaceum CR2. The sequencing data were deposited in the Sequence Read Archive (SRA) for CR1 with accession number SRR18220701 under BioProject accession number SAMN23410445, for CR2 with accession number SRR18220700 under BioProject accession number SAMN23391274, for CR3 with accession number SRR18220699 under BioProject accession number SAMN23410815, and for CR5 with accession number SRR18220698 under BioProject accession number SAMN23410818.

## References

[1] Leifson E. 1956. Morphological and physiological characteristics of the genus *Chromobacterium*. J Bacteriol 71:393–400. doi:10.1128/jb.71.4.393-400.1956. 13319251 PMC357816

[2] Hungria M, Astolfi-Filho S, Chueire LMO, Nicolás MF, Santos EBP, Bulbol MR, Souza-Filho A, Nogueira Assunção E, Germano MG, Vasconcelos ATR. 2005. Genetic characterization of *Chromobacterium* isolates from black water environments in the Brazilian Amazon. Lett Appl Microbiol 41:17–23. doi:10.1111/j.1472-765X.2005.01724.x. 15960747

[3] Benomar S, Evans KC, Unckless RL, Chandler JR. 2019. Efflux pumps in *Chromobacterium* species increase antibiotic resistance and promote survival in a coculture competition model. Appl Environ Microbiol 85:e00908-19. doi:10.1128/AEM.00908-19. 31324628 PMC6752006

[4] Shapiro-Ilan DI, Cottrell TE, Bock C, Mai K, Boykin D, Wells L, Hudson WG, Mizell RF. 2017. Control of pecan weevil with microbial biopesticides. Environ Entomol 46:1299–1304. doi:10.1093/ee/nvx144. 29028999

[5] Balusu RR, Fadamiro HY. 2012. Evaluation of organically acceptable insecticides as stand-alone treatments and in rotation for managing yellowmargined leaf beetle, *Microtheca ochroloma* (Coleoptera: Chrysomelidae), in organic crucifer production. Pest Manag Sci 68:573–579. doi:10.1002/ps.2297. 22231993

[6] Van Soolingen D, Embden PEW, de WH, va Hermans JDA. 1994. DNA fingerprinting of mycobacterium tuberculosis. Methods Enzymol 235:196–205. doi:10.1016/0076-6879(94)35141-4. 8057895

[7] De Oliveira NC, Rodrigues AA, Alves MIR, Filho NRA, Sadoyama G, Vieira JDG. 2012. Endophytic bacteria with potential for bioremediation of petroleum hydrocarbons and derivatives. Afr J Biotechnol 11:2977–2984.

[8] Tatusova T, DiCuccio M, Badretdin A, Chetvernin V, Nawrocki EP, Zaslavsky L, Lomsadze A, Pruitt KD, Borodovsky M, Ostell J. 2016. NCBI prokaryotic genome annotation pipeline. Nucleic Acids Res 44:6614–6624. doi:10.1093/nar/gkw569. 27342282 PMC5001611

[9] Li W, O'Neill KR, Haft DH, DiCuccio M, Chetvernin V, Badretdin A, Coulouris G, Chitsaz F, Derbyshire MK, Durkin AS, Gonzales NR, Gwadz M, Lanczycki CJ, Song JS, Thanki N, Wang J, Yamashita RA, Yang M, Zheng C, Marchler-Bauer A, Thibaud-Nissen F. 2021. RefSeq: expanding the Prokaryotic Genome Annotation Pipeline reach with protein family model curation. Nucleic Acids Res 49:D1020–D1028. doi:10.1093/nar/gkaa1105. 33270901 PMC7779008

[10] Kumar S, Stecher G, Li M, Knyaz C, Tamura K. 2018. MEGA X: molecular evolutionary genetics analysis across computing platforms. Mol Biol Evol 35:1547–1549. doi:10.1093/molbev/msy096. 29722887 PMC5967553

[11] Tamura K, Nei M. 1993. Estimation of the number of nucleotide substitutions in the control region of mitochondrial DNA in humans and chimpanzees. Mol Biol Evol 10:512–526. doi:10.1093/oxfordjournals.molbev.a040023. 8336541

[12] Jolley KA, Maiden MCJ. 2010. BIGSdb: scalable analysis of bacterial genome variation at the population level. BMC Bioinformatics 11:595. doi:10.1186/1471-2105-11-595. 21143983 PMC3004885

